# Journal of Suggestive Therapeutics

**Published:** 1898-11

**Authors:** 


JOURNAL OF SUGGESTIVE THERAPEUTICS. 211–212 Times-Herald Building, Chicago.

The second article on "How to Hypnotize" is the opening paper by the editor in the October number of *Suggestive Therapeutics* (Psychic Publishing Company, 211–212 Times-Herald Building, Chicago), in which the author seeks to demonstrate that there is no such thing as hypnotic control. A paper on "Healing During Natural Sleep," by Albert H. Burr, M. D., shows the theoretical principle of this method of treatment. Dr. H. L. True contributes his second article on the "Phenomena of Spiritualism," giving the purport of several "Messages" received in their circles, which are difficult of explanation. "The Relation of Hypnotism to Crime" is ably treated by M. Jules Liegeois, of the Faculty of Nancy, France, in a paper first read before the Congress at Moscow. This paper, which is now translated into English for the first time, is important as presenting the views of the School of Nancy upon this subject. "The Cure of Anemia by Suggestive Therapeutics" is explained by Dr. H. A. Parkyn, and the editor gives in detail his method of treating and curing the tobacco habit, expressing that method in two words—Suggestion and Peanuts! Editorial Notes and Answers to Correspondents complete a very valuable number.

